# A new option in the treatment of erectile dysfunction highligheted in International Brazilian Journal of Urology

**DOI:** 10.1590/S1677-5538.IBJU.2023.04.01

**Published:** 2023-08-07

**Affiliations:** 1 Universidade do Estado do Rio de Janeiro - Uerj Unidade de Pesquisa Urogenital Rio de Janeiro RJ Brasil Unidade de Pesquisa Urogenital - Universidade do Estado do Rio de Janeiro - Uerj, Rio de Janeiro, RJ, Brasil; 2 Hospital Federal da Lagoa Serviço de Urologia Rio de Janeiro RJ Brasil Serviço de Urologia, Hospital Federal da Lagoa, Rio de Janeiro, RJ, Brasil

The July-August number of *Int Braz J Urol* is the 23^nd^ under my supervision. In this number the Int Braz J Urol presents original contributions with a lot of interesting papers in different fields: Robotic Surgery, Prostate Cancer, Bladder Cancer, UPJ obstruction, Erectile Dysfunction, Urinary incontinence, Upper Tract Urothelial Carcinoma, Vasectomy, LUTS and Ejaculatory Physiology. The papers came from many different countries such as Brazil, Netherlands, Taiwan, USA and China, and as usual the editor´s comment highlights some of them. The editor in chief would like to highlight the following works:

Dr. Vieiralves and collegues from Brazil, presented in page 428 (1) a nice review, the cover of the present edition, about the low-intensity extracorporeal shockwave therapy (LIEST) for erectile dysfunction (ED) and concluded that literature presents little scientific evidence but suggests good results with the use of LIEST for ED. Despite a real optimism since it is a treatment modality capable of acting on the pathophysiology of ED, we must remain cautious, until a lar- ger volume of higher quality studies allows us to establish which patient profile, type of energy and application protocol will achieve clinically satisfactory results.

Dr. van Kollenburg and collegues from Netherlands, presented in page 411 (2) a important systematic review about the Novel minimally invasive treatments for lower urinary tract symptoms and concluded that five minimally invasive treatments (MITs) for treatment of LUTS were identified. Aquablation is likely to result in functional outcomes most comparable to TURP. Second in ranking was prostatic artery embolization, a technique that does not require general or spinal anesthesia. MITs have a better safety profile compared to TURP. However, due to high study heterogeneity, results should be interpreted with caution.

Dr. Carvalho and collegues from Brazil performed in page 452 (3) a nice study about the clinical and Urodynamic results of the Argus T^®^ sling in moderate and severe male stress urinary incontinence treatment and concluded that a long-term efficacy and safety of Sling Argus T^®^ as an alternative to moderate and severe male stress urinary incontinence treatment. Furthermore, in our study bulbar urethra compression does not lead to bladder outlet obstruction.

Dr. Gorgen and collegues form Brazil performed on page 462 (4) an interesting study about the laparoscopic pyeloplasty learning curve improvement by a standardized simulation training program and concluded that a structured laparoscopic simulation program can improve outcomes of laparoscopic pyeloplasty during the learning curve.

Dr. Huang and Collegues form Taiwan performed on page 469 (5) a nice study about the effects of Different Combinations of Radical Nephroureterectomy and Bladder Cuff Excision Procedures for Upper Tract Urothelial Carcinoma on Bladder Recurrence and concluded that patients with upper tract urothelial carcinoma, minimally invasive (MIS) radical nephroureterectomy (RNU) with open bladder cuff excision (BCE) is associated with a higher risk of intravesical recurrence than open RNU with open BCE and MIS RNU with intracorporeal BCE.

Dr. Geng and collegues from China performed on page 441 (6) a nice metanalysis about the effect of perioperative pelvic floor muscle exercise on urinary incontinence after radical prostatectomy and concluded that the application of Pelvic floor muscle exercise (PFME) after radical prostatectomy significantly reduced the incidence of early post operative urinary incontinence (UI) and additional preoperatively PFME had no significant improvement on the recovery of UI.

Dr. Baird and collegues from USA performed on page 479 (7) an study about the oncological Outcomes of Visibly Complete Transurethral Resection Prior to Neoadjuvant Chemotherapy for Bladder Cancer and concluded that A visibly complete transurethral resection of a bladder tumor (TURBT) was not associated with pathologic downstaging, cancer-specific or recurrence-free survival following neoadjuvant chemotherapy (NAC) and Radical Cystecomy (RC). These data do not support the need for repeat TURBT to achieve a visibly complete resection if NAC and RC are planned.

Dr. Lawton and collegues from USA performed on page 490 (8) a interesting study about the Risk of Post-Vasectomy Infections and concluded that Risk of infection after vasectomy is low, about 1%, among international high-volume vasectomy practices performing no- scalpel vasectomy and various occlusion techniques. Apart from vasectomy occlusion technique, no other factor modified the risk of post- vasectomy infection.

Dr. Westin and collegues from Brazil permorfed on page 501 (9) a nice report about a new surgical technique: bladder mucosal graft harvested with holmium: YAG (HO:YAG) laser - A new option in bulbar replacement urethroplasty and concluded that Transurethral harvesting of bladder mucosa using the Holmium laser (Ho-YAG) is feasible and reproducible. Our preliminary experience suggests that bladder mucosa grafts achieve comparable results to other grafts when used for dorsal onlay urethroplasty. Further research is needed to confirm these results.
